# Integrated genomic analysis reveals actionable targets in pediatric spinal cord low-grade gliomas

**DOI:** 10.1186/s40478-022-01446-0

**Published:** 2022-09-26

**Authors:** Misove Adela, Vicha Ales, Broz Petr, Vanova Katerina, Sumerauer David, Stolova Lucie, Sramkova Lucie, Koblizek Miroslav, Zamecnik Josef, Kyncl Martin, Holubova Zuzana, Liby Petr, Taborsky Jakub, Benes Vladimir, Pernikova Ivana, Jones T. W. David, Sill Martin, Stancokova Terezia, Krskova Lenka, Zapotocky Michal

**Affiliations:** 1 Prague Brain Tumor Research Group, Second Faculty of Medicine, Charles University and University Hospital Motol, Prague, Czech Republic; 2 Department of Pediatric Haematology and Oncology, Second Faculty of Medicine, Charles University and University Hospital Motol, Prague, Czech Republic; 3 Department of Pathology and Molecular Medicine, Second Faculty of Medicine, Charles University Prague and Faculty Hospital Motol, Prague, Czech Republic; 4 Department of Radiology, Second Faculty of Medicine, Charles University in Prague and Motol University Hospital, Prague, Czech Republic; 5 Department of Neurosurgery, Second Faculty of Medicine, Charles University and University Hospital Motol, Prague, Czech Republic; 6 Department of Neurology, Second Faculty of Medicine, Charles University and University Hospital Motol, Prague, Czech Republic; 7 Hopp Children’s Cancer Center Heidelberg (KiTZ), Heidelberg, Germany; 8 Pediatric Glioma Research Group, German Cancer Research Center (DKFZ), Heidelberg, Germany; 9 Division of Biostatistics, German Cancer Research Center (DKFZ), Heidelberg, Germany; 10 Department of Pediatric Oncology and Hematology, Children’s University Hospital, Banska Bystrica, Slovakia

**Keywords:** Spinal cord, Low-grade glioma, *KIAA1549:BRAF* fusion, *NTRK* fusion, Methylation profiling

## Abstract

Gliomas are the most common central nervous tumors in children and adolescents. However, spinal cord low-grade gliomas (sLGGs) are rare, with scarce information on tumor genomics and epigenomics. To define the molecular landscape of sLGGs, we integrated clinical data, histology, and multi-level genetic and epigenetic analyses on a consecutive cohort of 26 pediatric patients. Driver molecular alteration was found in 92% of patients (24/26). A novel variant of *KIAA1549:BRAF* fusion (*ex10:ex9*) was identified using RNA-seq in four cases. Importantly, only one-third of oncogenic drivers could be revealed using standard diagnostic methods, and two-thirds of pediatric patients with sLGGs required extensive molecular examination. The majority (23/24) of detected alterations were potentially druggable targets. Four patients in our cohort received targeted therapy with MEK or NTRK inhibitors. Three of those exhibited clinical improvement (two with trametinib, one with larotrectinib), and two patients achieved partial response. Methylation profiling was implemented to further refine the diagnosis and revealed intertumoral heterogeneity in sLGGs. Although 55% of tumors clustered with pilocytic astrocytoma, other rare entities were identified in this patient population. In particular, diffuse leptomeningeal glioneuronal tumors (n = 3) and high-grade astrocytoma with piloid features (n = 1) and pleomorphic xanthoastrocytoma (n = 1) were present. A proportion of tumors (14%) had no match with the current version of the classifier. Complex molecular genetic sLGGs characterization was invaluable to refine diagnosis, which has proven to be essential in such a rare tumor entity. Moreover, identifying a high proportion of drugable targets in sLGGs opened an opportunity for new treatment modalities.

**Supplementary Information:**

The online version contains supplementary material available at 10.1186/s40478-022-01446-0.

## Introduction

The majority of CNS tumors are located intracranially, and only 5% occur in the spinal cord [1]. Intramedullary spinal cord tumors have a glial origin and are biologically low-grade in 95%. Similarly, as in brain low-grade gliomas (LGGs), spinal cord (= intramedullary) low-grade gliomas in children (sLGGs) show a chronic course of the disease affecting the quality of life while the overall survival remains excellent [2]. Treatment of choice is maximal safe surgical resection under intraoperative monitoring which must not endanger neurological function as chemotherapy can stabilize the progression of the disease in 40–50% [3, 4].

The urge is to predict the risk of progression and search for novel, effective treatment approaches. Molecular data are seldom, suggesting that the most prevalent alteration is *KIAA1549:BRAF* fusion [5]. Due to the rarity of the disease, genomic data are scarce or incomplete, and the use of targeted therapy in sLGGs was not demonstrated yet. Therefore, an institutional integrated clinical and comprehensive genetic study was conducted to reveal sLGG-associated molecular alterations and their therapeutic implications, assess intertumoral heterogeneity using methylation profiling, and demonstrate the effect of targeted therapy in this group of patients.

## Methods

### Patient cohort and clinical follow-up

Tumor samples and clinical data from patients with sLGGs diagnosed and treated at our center from 2000 to 2021 were retrieved for survival, radiologic data, and molecular evaluation. Patients were followed on an out-patient or in-patient basis with regular MRI imaging. All clinical data were collected retrospectively. The last patient was enrolled on 02/11/2020, and the disease status for all patients was updated on 01/07/2021. The institutional review board approved the study, and all patients obtained informed consent as per our routine procedure.

### Volumetric analysis

Volumetric analysis was implemented to measure tumor response to the targeted therapy. Lesions' volumes were estimated with open-source software 3D Slicer (version 4.10.2) using basic modules (Segment Editor, Segment Statistics) [6]. MRI sequences with the best spatial and contrast resolution were selected from available examinations for lesion volume evaluation.

### DNA and RNA extraction

The nucleic acids were extracted from formalin-fixed, paraffin-embedded tissue block using QIAamp DNA FFPE Tissue Kit (Quiagen, Germany) for genomic DNA and using high pure RNA paraffin kit (Roche Diagnostics, Mannheim, Germany) for total RNA. In the case of fresh frozen sections, extraction of genomic DNA and total RNA was manufactured using Trizol Reagent (Life Technologies, Merelbeke, Belgium). Neuropathologists selected the most representative tissue blocks containing the maximum percentage of tumor tissue before isolation of nucleic acid.

### cDNA synthesis and conventional RT-PCR

cDNA was prepared from RNA (Life Technologies, Carlsbad, CA) according to the manufacturer's instructions. cDNA was subjected to conventional RT-PCR amplification with primers specific for common *KIAA1549:BRAF* variants(*ex16:ex9*, *ex15*:*ex9*, *ex16:ex11*) as previously described [7].

### Sanger sequencing

PCR and Sanger sequencing were conducted to examine hotspot mutations at codons 27 and 34 of *H3F3A*, codon 600 of *BRAF* ex15, codons 546 and 656 of *FGFR1* ex12, and codon of *FGFR1* ex14 using previously described primer pairs. Amplification was performed using 2 × PCRBIO HS Taq Mix Red (PCR Biosystems Ltd., London, UK). The PCR products were electrophoresed in a 1.5% agarose gel and were recovered using the Gel DNA Fragments Extraction Kit (Geneaid, Taiwan). Sanger sequencing was performed using Big Dye Terminator v 3.1 chemistry (LifeTechnologies) and an ABI PRISM 3130 genetic analyzer Applied Biosystems. Results were analyzed using Chromaslite 2.01 (Technelysium, PtyLtd, Brisbane, Australia).

### MLPA

SALSA® MLPA® probemix P370 can be used to detect genomic duplications leading to the *KIAA1549:BRAF*, *SRGAP3:RAF1*, and *FGFR1:TACC1* fusion genes and for detection of copy number aberrations in the *BRAF, CDKN2A/2B, FGFR1, MYB*, and *MYBL1* genes. Furthermore, this probemix contains five specific probes detecting the BRAF p.V600E & four predominant IDH1 p.R132H and p.R132C and IDH2 p.R172M and p.R172K point mutations, which will only generate a signal when the mutation is present. MLPA was performed following the manufacturer's instructions. Data were analyzed using Coffalyser Software (MRC-Holland, Amsterdam, The Netherlands).

### RNA panel sequencing

Based on the quality of preserved nucleic acid in the samples, we were using one of Archer® FusionPlex® (Archer DX, Boulder, Colorado) commercially available panels—either Lung kit or Oncology Research kit. Despite different nucleic acid quality requirements resulting from the different number of analyzed genes, both panels are used for fusion and SNV identification with the advantage of identifying the fusion even with an unknown partner. Archer® FusionPlex® also offers robust performance even for FFPE samples. RNA extraction, library preparation, and parallel sequencing were performed as per the manufacturer's recommendation. Anchored Multiplex polymerase chain reaction amplicons were sequenced on Illumina MiSeq, and the data were analyzed using the Archer and Arriba [8] (https://github.com/suhrig/arriba/) softwares. RT-PCR was performed to validate the fusion transcript identified by RNA sequencing (Additional file 1: Table S1).

### Methylation profiling

DNA methylation was performed using the Infinium Methylation EPICBeadChip Kit (Illumina, San Diego, CA, USA). A total of 250 ng of DNA from fresh frozen tumor tissue was treated with bisulfite conversion using the ZymoResearch EZ DNA Methylation kit (Zymo Research Corp, Irvine, CA, USA). In the case of FFPE samples, DNA restoration was performed as per the manufacturer's instructions (Infinium FFPE DNA Restoration kit, Illumina, San Diego, CA, USA). According to the manufacturer's explicit specifications, the Infinium HD Methylation Assay was performed at the laboratories of the Department of Pediatric Haematology and Oncology, Second Faculty of Medicine in Prague. The methylation class was established using web-based analysis via https://www.molecularneuropathology.org/ using publically available v11b4 and v12.5 versions of brain classifier. To compare spinal cord glioma samples with the DKFZ reference cohort, t-SNE analysis was performed with 10,000 most differentially methylated probes using Rtsne package v.0.13 as previously described [9].

### Statistical analysis

Progression-free survival and overall survival were analyzed by the Kaplan–Meier method, and p-values reported using the log-rank test in an open-source R statistical environment (v4.1.2), using R packages survival (v2.41–3), and ggplot2 (v2.2.1).

## Results

### Patient selection

**Fig. 1:**
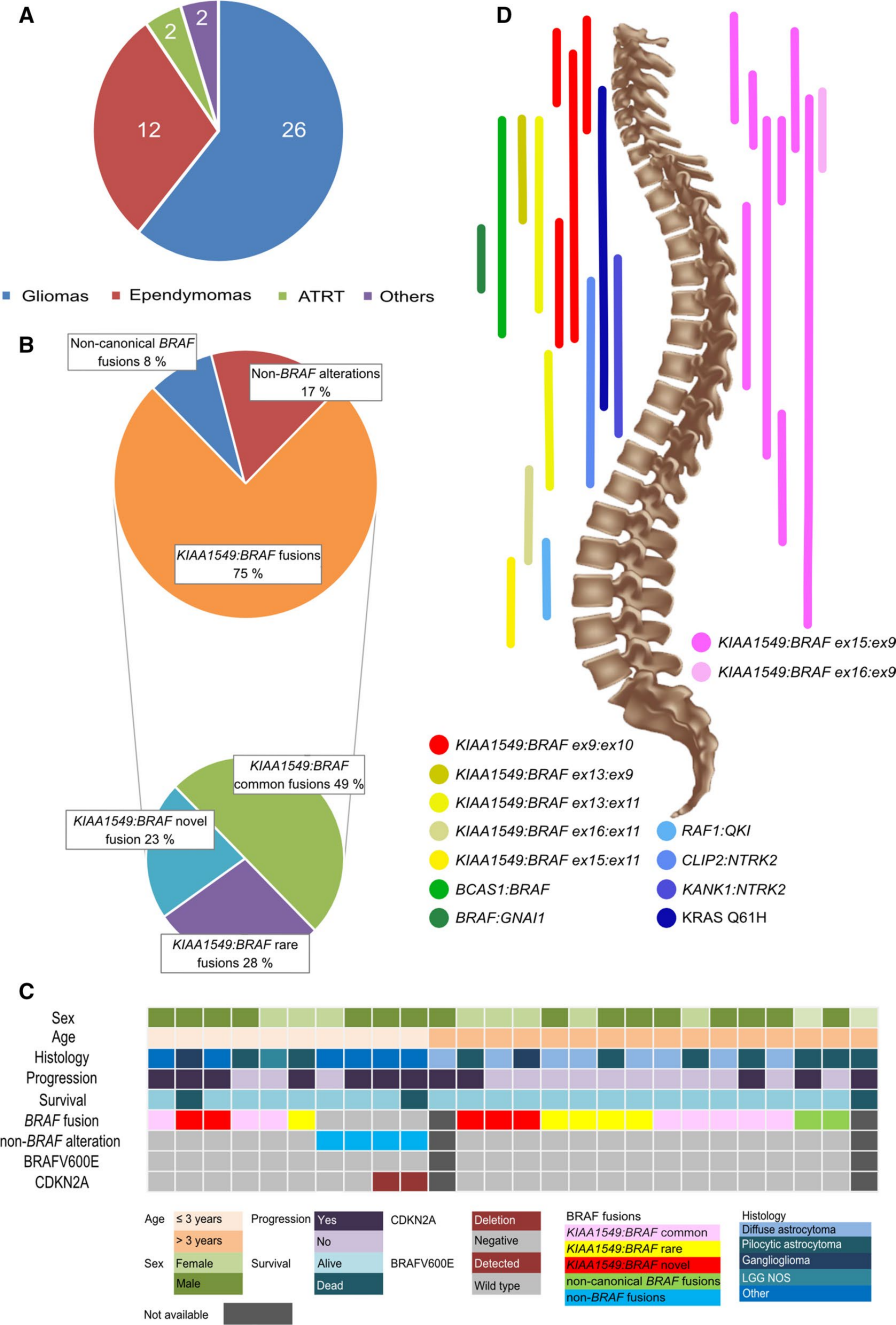
**a** Overview of the total number of 42 spinal cord tumors diagnosed within 2000–2021. **b** Pie of pie demonstrates three molecular alteration groups in sLGGs; tumors driven with canonical *BRAF* fusions, non-canonical *BRAF* fusions, and non-*BRAF* alterations. **c** Oncoplot summarizes the relation of demographic (sex, age), clinical (progression, survival), and molecular-pathology data (histology, driver alteration, *CDKN2A* status), **d** Comparison of the molecular alterations, anatomical location, and extent of the tumors. The position of vertical lines shows the anatomical location of the tumor and the length of vertical lines outlines the levels of the spinal cord affected by each tumor sample. Molecular subtypes are shown in colors. On the left side are displayed common *KIAA1549:BRAF* fusions (pink) in contrast with the right side where are rare *KIAA1549:BRAF* fusions (yellow), a novel type of *KIAA1549:BRAF* fusion (red), non-*KIAA1549:BRAF* fusions (green) and non-*BRAF* alterations (blue)

During the study period, 42 pediatric patients with spinal cord tumors were diagnosed, excluding non-biopsied patients with known Neurofibromatosis type 2. Diagnosis of sLGGs was made in 23 patients; 12 patients were diagnosed with ependymoma, and four patients with ATRT or other entity (Fig. 1a). Retrospective evaluation of the spinal cord tumor cohort revealed discordance between histology and the clinical course of the disease in three patients. Additional testing done in 2 ependymoma patients with atypical clinical course revealed rather glial tumors with ependymal features. Furthermore, one long-term surviving patient with an inoperable tumor initially described as anaplastic astrocytoma showed rather low-grade biology of the tumor with multiple progressions over many years of follow-up. In this particular patient, molecular studies revealed *NTRK2* fusion and confirmed a glial origin of the tumors with low-grade behavior. Therefore, these three patients were added to the sLGGs group in this article to a total number of 26 patients (Additional file 2: Table S2; sLGG_01—sLGG_26), representing 7% of all institutional pediatric LGGs (total number = 350). Median age at diagnosis for the sLGGs group was 4.55 years (range from 1.15 to 17.54 years). Histologically, the sLGGs group comprised predominantly of pilocytic astrocytoma, diffuse astrocytoma, and ganglioglioma.

### Comprehensive genomic analysis uncovered a novel and rare alterations within the MAPK pathway

Comprehensive genetic analysis consisting of Sanger sequencing, MLPA, and RNA sequencing was employed to determine the genetic landscape of pediatric LGGs. Oncogenic driver alterations were detected in 93% (n = 24) of patients; no alterations were detected in two patients where analysis failed due to insufficient tissue quality. Alterations found across sLGGs could be classified into two groups (Fig. 1b: 1) *BRAF* alteration consisting of *KIAA1549:BRAF* fusions (75%) with the high occurrence of rare and novel fusion variants, and non-KIAA1549:*BRAF* fusions (8%), 2) non-*BRAF* alterations (17%). Surprisingly, no case harboring BRAFV600E or *H3F3A/HIST1H3B* mutation was identified. We also evaluated the presence of the secondary alterations and found two cases of *CDKN2A* homozygous deletion in non-*BRAF* tumors. (Fig. 1c) Furthermore, pathogenic *MET* and *EGFR* variants were detected in two *KIAA1549:BRAF* cases.

**Fig. 2:**
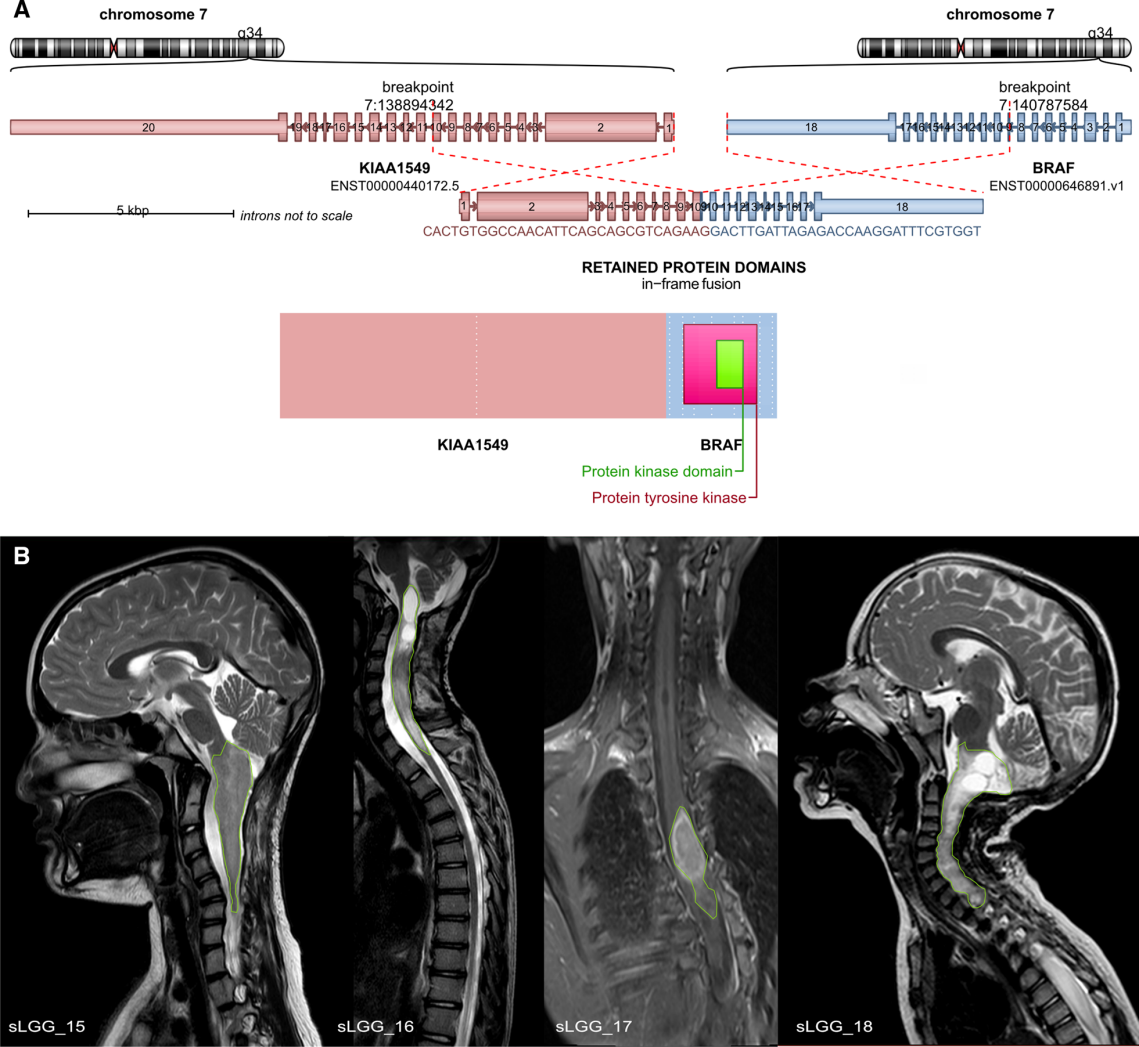
**a** Novel *KIAA1549:BRAF* fusion variant—*ex9:ex10*. The diagram shows an in-frame fusion gene incorporating the kinase domain of *BRAF* oncogene. **b** MRI images demonstrate similarities in the anatomical location of sLGGs in patients with detected novel *KIAA1549:BRAF ex9:ex10* fusion variant. Tumors are delineated with green line

A novel variant of *KIAA1549:BRAF* fusion (*ex10:ex9*) was identified using RNA sequencing in four cases (Fig. 2a). Rare types of *KIAA1549:BRAF* fusions (*ex13:ex9, ex13:ex11, ex16:ex11, ex15:ex11*) were identified in further 21% sLGG patients. Non-canonical *BRAF* fusions were detected in two patients; accounting for *BCAS1:BRAF* and *GNAI1:BRAF*. Two independent methods verified those rare and novel *BRAF* fusions using RT-PCR with specific primers and chromosome 7q34 duplication using MLPA.

Anatomical distribution of the genetic alterations (Fig. 1d) interestingly showed *KIAA1549:BRAF ex10:ex9*-positive tumors located in the upper half of the spinal cord with partial medulla oblongata involvement in two cases (two cases in the cervical spine (C1–C7), one case in the cervical and upper thoracic spine (C2–T2), and one in the upper thoracic spine (T2–T7) (Fig. 2b). To evaluate the frequency of the *KIAA1549:BRAF ex10:ex9* fusion variant, the cohort was expanded using 205 institutional cases of intracranial pediatric LGGs of various locations with known molecular drivers (Additional file 3: Fig. S1). Among more than 50 cases with detectable *KIAA1549:BRAF*, no case harbored an *ex10:ex9* variant suggesting exclusive occurrence in the spinal cord.

Non-*BRAF* alterations were detected in four tumor samples consisting of *CLIP2:NTRK2*, *KANK1:NTRK2*, *RAF1:QKI*, and KRAS Q61H. The young age of three years and under at diagnosis characterized this group of patients. This group's histological appearance was not typical for LGG, and molecular testing helped refine the diagnosis. *CLIP2:NTRK2* case was diagnosed as an anaplastic astrocytoma grade 3. Despite HGG histology, the presence of *CDKN2A* homozygous deletion, and multiple progressions, the patient was alive 15 years from the time of diagnosis. Pathologists reported two cases (*KANK1:NTRK2* and *RAF1:QKI*) as ependymomas, but these tumors were reclassified as low-grade glioma due to the clinical course, underlying molecular alteration, and methylation profile. Based on the molecular profile, the case with *KANK1:NTRK2* that also harbored *CDKN2A* homozygous deletion was treated with a radiation-sparing approach using chemotherapy only as first-line therapy. Patient with sLGG harboring *RAF1:QKI* underwent subtotal resection followed by careful observation. KRAS Q61H mutated case had histology of low-grade glioneuronal tumor (LGNT), and the patient was observed only after partial resection.

### Methylation profiling revealed significant intertumoral heterogeneity among sLGGs

**Fig. 3:**
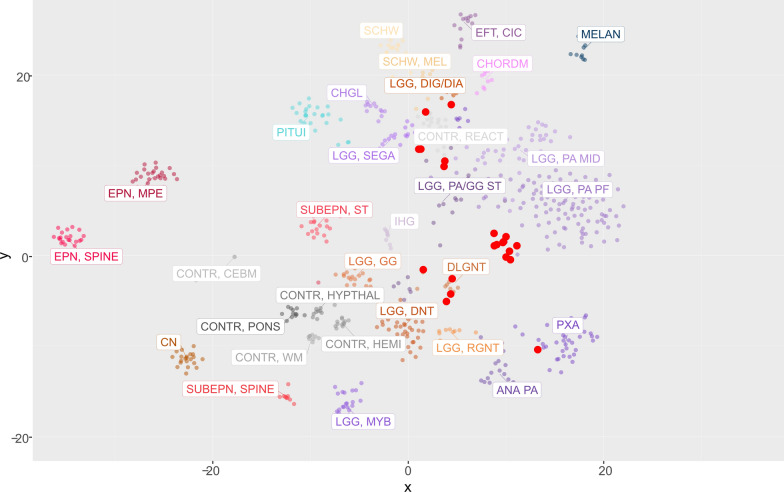
T-SNE plot demonstrates the intertumoral heterogeneity among sLGGs. Prague samples (large red dots) are displayed among cases relevant methylation classes. This figure displays a close-up of the t-SNE with the whole reference cohort (see Additional file 4: Fig S2)

The current version (v12.5) of the Heidelberg methylation classifier does not provide any methylation class specific for spinal cord gliomas in contrast to spinal cord ependymomas. Therefore, we performed methylation profiling to evaluate how would sLGGs be classified based on the epigenetic features and to discern intertumoral heterogeneity. The analysis was performed using publically available Heidelberg classifier v12.5. Out of 22 patients (91.6%) with sufficient tissue available, 12 tumors (55%) were predicted as pilocytic astrocytoma, subclass posterior fossa (PA-PF) despite variable calibrated scores (calibrated scores (CS) 0.35–0.99). Three tumors (14%) with 1p deletion matched with diffuse leptomeningeal glioneuronal tumor (DLGNT), methylation class 1 (DLGNT – MC1) (two CS 0.99, one CS 0.25). One anaplastic astrocytoma with *CLIP2:NTRK2* fusion was classified as anaplastic pilocytic astrocytoma (CS 0.62), currently also known as high-grade astrocytoma with piloid features (HGAP). The other *NTRK2* fused glioma (*KANK1:NTRK2*), originally diagnosed as ependymoma, was classified as pleomorphic xanthoastrocytoma (PXA) (CS 0.90). One case (*QKI:RAF1*) was clustered with a subtype A of glioneuronal tumors (CS 0.99). One case (*KIAA1549:BRAF ex10:ex9*) was classified as desmoplastic infantile ganglioglioma / desmoplastic infantile astrocytoma (CS 0.59). The remaining three tumors (14%) matched with control tissue most probably due to low tumor tissue content. Moreover, t-SNE analysis using a previously published reference cohort was performed to further refine the methylation class prediction. As classified by v12.5, significant proportion of the samples clustered nearby PA-PF cluster. They seemed to be forming a separate cluster suggesting a possible distinction from posterior fossa pilocytic astrocytoma. Remaining samples clustered with DLGNT, GNT, PXA, and other clusters as predicted with the classifier (Fig. 3).

### Clinical outcome and exploitation of molecular targets

**Fig. 4:**
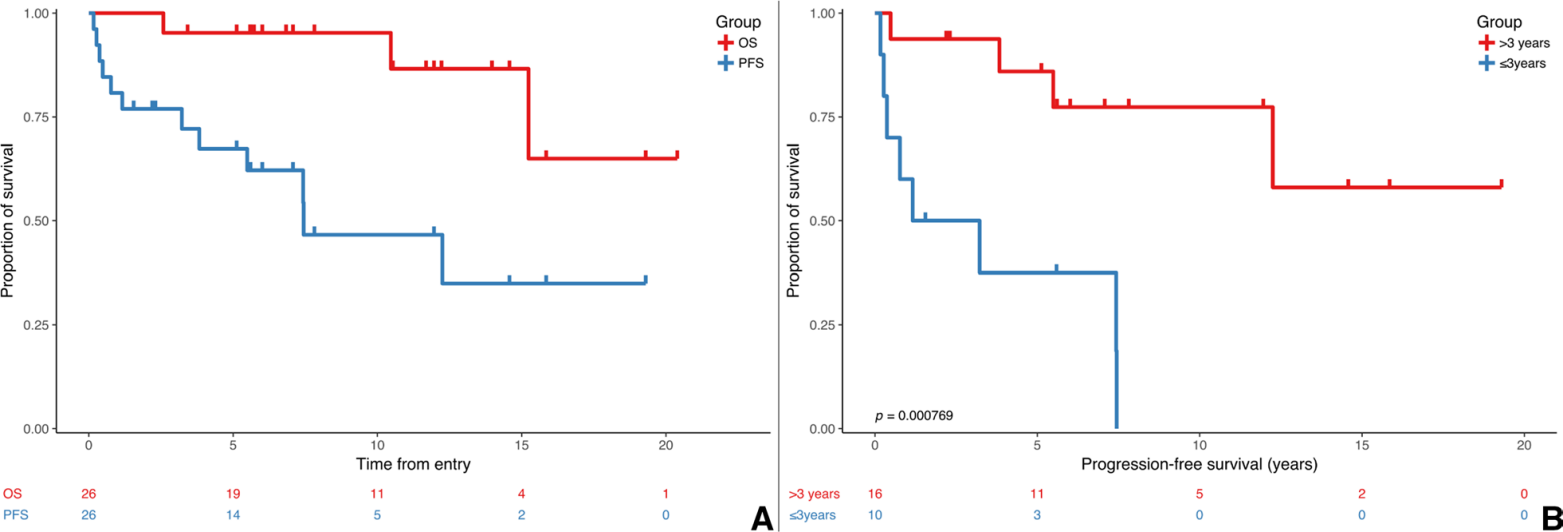
Kaplan Meier survival analysis. **a** PFS and OS of the cohort. **b** Significantly worse PFS 37.5% (CI 95%, 16.2–86.8%) of younger children compared to PFS 85.9% (CI 95%, 69.5 to 100%) in older children (*p* < 0.001)

At a median follow-up of 6.96 years (IQR: 3.42–12.22), 5-year progression-free survival was 67.3% (95% confidence interval [CI], 50.8–89.1%). Overall survival rate at 5 years was 95.2% (95% [CI], 86.6–100.0%) (Fig. 4a). Infants of three years and younger fared significantly worse compared to those older than three with 5-year PFS 37.5% (CI 95%, 16.2–86.8%) and 85.9% (CI 95%, 69.5–100%), respectively (*p* < 0.001) (Fig. 4b) Three patients died of the disease 15, 10, and two years after diagnosis respectively due to the progressive disease, regardless of the histological grade or molecular alteration (one with *CLIP2:NTRK2*, one without known driver alteration, and one with *KIAA1549:BRAF ex10:ex9* variant).

**Fig. 5:**
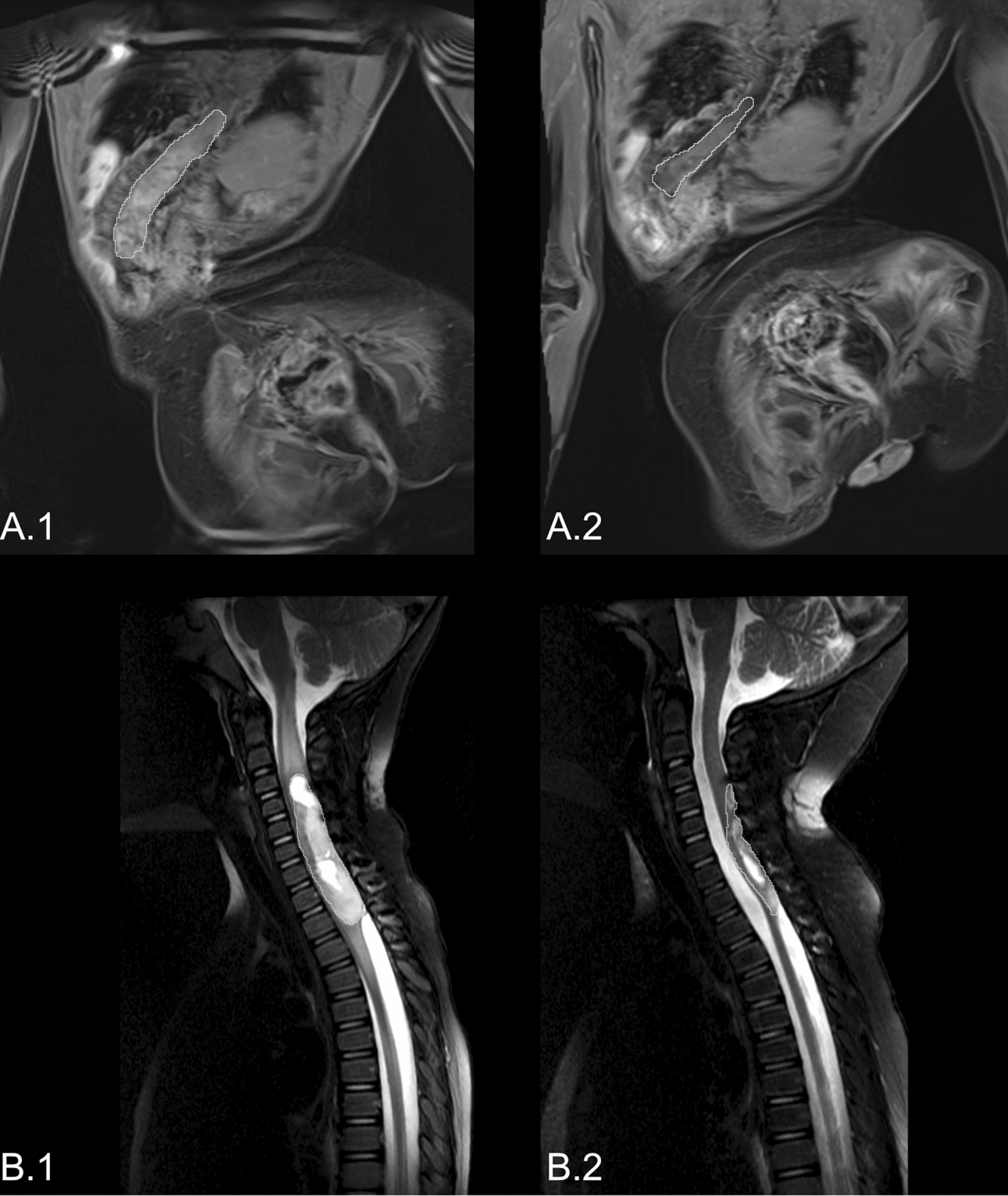
Radiological response to the targeted therapies. The white line (**a.1**) indicates tumor volume before initiation of NTRK inhibitor in patients sLGG_22, and (**a.2**) shows a 40% tumor volume reduction after 54 days of therapy. Likewise, the white line (**b.1**) shows tumor volume before initiation of MEK inhibitor in patient sLGG_07, and (**b.2**) shows a 61% tumor volume reduction after eight months of therapy

Based on molecularly identified targets, four patients received targeted therapy using MAPK pathway inhibitors. Three patients with *KIAA1549:BRAF* fusion were treated with MEK-inhibitor trametinib. According to the volumetric measurements, one of the patients (sLGG_15) responded with stable disease, and two patients (sLGG_06 and sLGG_07) exhibited partial responses (reduction of 51% and 61%) that were achieved after 5 and 8 months, respectively. Patient (sLGG_22) with *CLIP2:NTRK2* fusion was treated with TRK inhibitor larotrectinib with quickly induced volume reduction, not meeting partial response (40%), detectable on magnetic resonance 54 days after the therapy initiation (Fig. 5). The radiological volume reduction was accompanied by significant clinical improvement with no remarkable drug-related toxicity. Unfortunately, tumor progression accompanied by clinical decline was detected after 22 months of targeted therapy. After another five months, the patient died due to the tumor progression to the brainstem. DNA sequencing from autopsy material did not reveal any point mutations in the *NTRK1/2/3* kinase domain; thus, the mechanism of acquired resistance remained unknown [10].

## Discussion

Here we present a study with comprehensive genomic and epigenomic analysis in a 20-year retrospective single institutional non-selected cohort of 26 consecutive sLGG patients in the context of clinical course, including quantitative imaging analysis and employment of novel treatment modalities. New findings regarding significant epigenetic heterogeneity were for the first time reported in pediatric sLGG population. Furthermore, identification of fusion landscape including novel fusion variants highlighted the impact of our data on diagnostics and new therapeutic opportunities.

We confirmed that the majority of pediatric sLGGs harbored *KIAA1549:BRAF* fusions, but unlike intracranial location, rare and novel variants were predominantly present. In particular, the novel *KIAA1549:BRAF ex10:ex9* was uncovered in our study. Importantly, this fusion variant was not found in any of our institutional pediatric intracranial LGGs cases, making this variant specific for the spinal cord compartment. Moreover, to our knowledge, this fusion variant has not been reported yet in the literature. As reported in other *KIAA1549:BRAF* variants, the variant *ex10:ex9* also lacked the autoinhibitory domain and caused MAPK activation in the same manner [11, 12] (Fig. 2a).

Non-*BRAF* fusions were detected in children younger than three years and consisted of tumors with *NTRK2* and *RAF1* fusions and *KRAS* mutation. The clinical course of the disease in non-*BRAF* fusion patients tended to progress requiring multiple treatment modalities. Furthermore, two patients with non-canonical *BRAF* fusions (*BCAS1:BRAF*, *GNAI1:BRAF*) were revealed and confirmed that non-canonical *BRAF* fusions could occur in the sLGGs. Interestingly, there was no spinal cord glioblastoma characterized by histone *H3F3A* mutation in the presented cohort of spinal cord tumors [13]. No BRAF V600E mutation was detected in our cohort, probably due to a limited number of patients and a low prevalence of BRAF V600E mutation in sLGG [5, 14].

Survival in regards to 5-year PFS (67.3%) and OS (95.2%) was comparable with more extensive series with predominantly intracranial pediatric LGGs or sLGGs [5, 14]. Importantly, significantly worse PFS in younger children compared to the older ones stood out in the presented cohort. The extent of resection did not influence our PFS data as only two patients had their sLGGs completely removed. Therefore, the poorer outcome of younger children might have been related to the presence of non-*BRAF* alterations in combination with more frequent occurrence of cases with atypical histology. Our observation of a poorer prognosis was consistent with previously published clinical trials where young children fared significantly worse [15, 16]. Frequent non-*BRAF* fusions in younger children with sLGGs and histology not typical for true pediatric LGGs resembled cases of infant hemispheric gliomas, [17, 18] underlying the necessity of multi-layer diagnosis integrating histopathology and molecular genetics analysis. Some of our very young children with sLGGs were diagnosed with "ependymoma-like" histology, and further molecular investigation helped to refine the diagnosis.

Previously published studies evaluated the genomic landscape of spinal cord gliomas. More extensive series usually presented limited information on molecular alterations restricted to data from molecular biology methods such as FISH, NanoString platform, or RT-PCR [5], most probably due to poor tumor tissue availability. Grob et al. showed that 42% of sLGGs tested positive for *KIAA1549:BRAF* fusion [5]. It is essential to underline that the most frequent alterations in our cohort were rare and novel *KIAA1549:BRAF* fusion variants. The mechanism of *BRAF* activation remains the same, but commonly used diagnostic methods (RT-PCR with specific primers/NanoString) will not detect rare fusion variants, potentially compromising an opportunity for targeted therapy for those patients. The rare *KIAA1549:BRAF ex13:ex11* fusion variant was only described in one case of spinal glioneuronal tumor [19]. *RAF:QKI1* is known to activate MAPK/ERK and PI3K/mTOR signaling and was already described in a single case report of an adult diagnosed with spinal pleomorphic xanthoastrocytoma [20]. Moreover, some other larger studies focused on single nucleotide variants rather than gene fusions in the adult population and therefore were unable to capture the whole landscape of fusions related to pediatric sLGG [21, 22].

Methylation profiling using Heidelberg classification [9] was demonstrated as a powerful research tool in pediatric CNS tumors. We employed the whole-genome DNA methylation array to uncover epigenetic features of sLGGs. A dominant cluster with the methylation class "pilocytic astrocytoma, posterior fossa" suggested a possibility of a common cell of origin with the most frequent intracranial group of LGGs. Nevertheless, the current classifier scored 55% of tumors in our sLGGs group between 0.3 and 0.97. This was in keeping with t-SNE analysis demonstrating these tumors form a cluster nearby the PA-PF cluster. This data could suggest distinct epigenetic features of sLGGs compared to PA-PF. Moreover, tumors clustering with rare methylation classes were detected, in particular DLGNT, HGAP, and PXA. Three DLGNT cases were circumscribed as spinal cord lesions without any evidence of dissemination through the neuroaxis. HGAP and PXA were represented by cases with uncharacteristic histology (not clear LGGs), either anaplastic astrocytoma or anaplastic ependymoma, presence of *NTRK2* fusions, and long-term survival. Several cases (n = 3) did not match any methylation classes suggesting either high normal tissue content or a rare entity yet to be defined [23]. Previous study attempted to characterize spinal cord gliomas using methylation profiling. The cohort consisted of 19 adults and seven children with high-grade and low-grade gliomas, and therefore a portion of cases clustered with Diffuse Midline Glioma *H3FA3*-positive and glioblastoma *IDH* wild-type. They also described two adult cases with HGAP and very short survival, two cases with IDH mutant glioma, and one DLGNT. Eight cases matched with pilocytic astrocytoma, but the tissue was not available to perform RNA sequencing, and therefore authors were not able to demonstrate the presence of characteristic fusions [24].

Comprehensive genomic analysis was critical not only to uncover the molecular landscape of pediatric sLGGs but also to identify high-priority targets for novel therapies. In particular, targeted therapy was used in four sLGGs patients with progressive disease who presented with neurological decline. Previously, MEK-inhibitor trametinib was shown to benefit a proportion of patients with progressive NF1 or *BRAF*-driven LGGs [25, 26], and our series of cases suggest clinical benefit with objective responses documented on magnetic resonance imaging in sLGGs. In addition, NTRK inhibitors have shown high efficacy in multiple NTRK-driven cancers [27], and our case demonstrated significant clinical benefit of such therapy in the patient with *NTRK2* fused sLGG.

A relatively small cohort size, short follow-up, and retrospective data collection did not allow a more comprehensive prognostic marker evaluation. Furthermore, volumetry was used as a method for response evaluation considering bidimensional measurement less feasible [28], especially in our sLGG patients frequently suffering from significant spinal deformities. Therefore, response assessment in Pediatric Neuro-Oncology (RAPNO) was not implemented in this study [29].

Nevertheless, we have assembled a coherent group of solely pediatric patients with extensive molecular analysis, and our data demonstrated the importance of integrative molecular-pathological diagnosis and enlightening the potential of targeted treatment for sLGGs. Future large prospective studies evaluating prognostic markers, the efficacy of targeted therapies, and volumetric response assessment in sLGG are needed.

## Conclusion

This study provides essential data on the molecular background of purely pediatric cohort of anatomically defined low-grade gliomas confined to the spinal cord. Methylation profiling revealed epigenetic landscape of sLGG demonstrating that 55% of cases cluster with posterior fossa pilocytic astrocytoma samples with the remaining 45% being very heterogeneous. Despite this epigenetic heterogeneity, sLGGs harbor driver alterations within MAPK pathway. In contrast to the intracranial LGGs, non-*BRAF* fusions (including *NTRK* fusions) and rare *KIAA1549:BRAF* variants, including novel variant *ex9:ex10*, represent frequent molecular drivers in sLGG. Our data clearly demonstrated the presence of druggable targets, and our case series demonstrated promising results in disease control with targeted therapy. However, further research and prospective clinical trials are required to evaluate the role of targeted therapy in sLGG patients.

## Supplementary Information

**Additional file 1: Table S1**. Table of primers used to validate the fusion transcripts identified by RNA sequencing.

**Additional file 2: Table S2**. Table showing the complete sLGGs cohort emphasizing the original histology before molecular pathology reevaluation, anatomical location, and molecular-biology data.

**Additional file 3: Fig. S1**. A total number of pediatric intracranial LGG patients with known genetic alteration is dividedby anatomical location. Importantly, *KIAA1549:BRAF ex9:ex10* variant fusion was detected solely in the upperspine.

**Additional file 4: Fig. S2**. T-SNE analysis displaying Prague samples (large red dots) among reference cohort samples.

## Data Availability

The datasets are publically available at Mendeley Data https://doi.org/10.17632/xzkgt4jvm2.1.

## Abbreviations

ATRT: Atypical teratoid rhabdoid tumor
cDNA: Complementary DNA
CI: Confidence interval
CS: Calibrated score
DLGNT: Diffuse leptomeningeal glioneuronal tumor
DNA: Deoxyribonucleic acid
Ex: Exon
FGFR: Fibroblast growth factor receptors
GNT: Glioneuronal tumor
HGAP: High-grade astrocytoma with piloid features
IDH: Isocitrate dehydrogenase
IQR: Interquartile range
LGG: Low-grade glioma
LGNT: Low-grade glioneuronal tumor
MAPK: Mitogen‑activated protein kinase
MC: Methylation class
MEK: Mitogen-activated protein kinase kinase
MLPA: Multiplex ligation-dependent probe amplification
MRI: Magnetic resonance imaging
NTRK: Neurotrophic tyrosine receptor kinase
PA-PF: Pilocytic astrocytoma–posterior fossa
PCR: Polymerase chain reaction
PXA: Pilomyxoid astrocytoma
RAPNO: Response assessment in Pediatric Neuro-Oncology
RNA: Ribonucleic acid
RT-PCR: Reverse transcription polymerase chain reaction
sLGGs: Spinal low-grade gliomas
