# Correction to: Integrated genomic analysis reveals actionable targets in pediatric spinal cord low-grade gliomas

**DOI:** 10.1186/s40478-022-01467-9

**Published:** 2022-11-11

**Authors:** Adela Misove, Ales Vicha, Petr Broz, Katerina Vanova, David Sumerauer, Lucie Stolova, Lucie Sramkova, Miroslav Koblizek, Josef Zamecnik, Martin Kyncl, Zuzana Holubova, Petr Liby, Jakub Taborsky, Vladimir Benes, Ivana Pernikova, David T. W. Jones, Martin Sill, Terezia Stancokova, Lenka Krskova, Michal Zapotocky

**Affiliations:** 1 Prague Brain Tumor Research Group, Second Faculty of Medicine, Charles University and University Hospital Motol, Prague, Czech Republic; 2 Department of Pediatric Haematology and Oncology, Second Faculty of Medicine, Charles University and University Hospital Motol, Prague, Czech Republic; 3 Department of Pathology and Molecular Medicine, Second Faculty of Medicine, Charles University Prague and Faculty Hospital Motol, Prague, Czech Republic; 4 Department of Radiology, Second Faculty of Medicine, Charles University in Prague and Motol University Hospital, Prague, Czech Republic; 5 Department of Neurosurgery, Second Faculty of Medicine, Charles University and University Hospital Motol, Prague, Czech Republic; 6 Department of Neurology, Second Faculty of Medicine, Charles University and University Hospital Motol, Prague, Czech Republic; 7 Hopp Children’s Cancer Center Heidelberg (KiTZ), Heidelberg, Germany; 8 Pediatric Glioma Research Group, German Cancer Research Center (DKFZ), Heidelberg, Germany; 9 Division of Biostatistics, German Cancer Research Center (DKFZ), Heidelberg, Germany; 10 Department of Pediatric Oncology and Hematology, Children’s University Hospital, Banska Bystrica, Slovakia

**Correction to**: *Acta Neuropathologica Communications* (2022) 10:143 

10.1186/s40478-022-01446-0

Following publication of the original article [1], the authors identified an error in the author names. The given names and family names of all authors were erroneously transposed.

The incorrect author names are: Misove Adela, Vicha Ales, Broz Petr, Vanova Katerina, Sumerauer David, Stolova Lucie, Sramkova Lucie, Koblizek Miroslav, Zamecnik Josef, Kyncl Martin, Holubova Zuzana, Liby Petr, Taborsky Jakub, Benes Vladimir III, Pernikova Ivana, Jones T. W. David, Sill Martin, Stancokova Terezia, Krskova Lenka and Zapotocky Michal

The correct author names are: Adela Misove, Ales Vicha, Petr Broz, Katerina Vanova, David Sumerauer, Lucie Stolova, Lucie Sramkova, Miroslav Koblizek, Josef Zamecnik, Martin Kyncl, Zuzana Holubova, Petr Liby, Jakub Taborsky, Vladimir Benes III, Ivana Pernikova, David T. W. Jones, Martin Sill, Terezia Stancokova, Lenka Krskova and Michal Zapotocky

The author group has been updated above and the original article [1] has been corrected.

## References

[1] Misove (2022). Integrated genomic analysis reveals actionable targets in pediatric spinal cord low-grade gliomas. Acta Neuropathol Commun.

