# Supplementary material for: Integrated genomic analysis reveals actionable targets in pediatric spinal cord low-grade gliomas
Source: Acta Neuropathol Commun. 2022 Sep 26;10:143. doi: 10.1186/s40478-022-01446-0 (PMC9513869; doi:10.1186/s40478-022-01446-0)
Supplement: Supplementary file 1 — Additional file 1: Table S1. Table of primers used to validate the fusion transcripts identified by RNA sequencing. [file 40478_2022_1446_MOESM1_ESM.docx]

| Gene | Primer | Sequence (5´-3´) |
| --- | --- | --- |
| KIAA1549  exon13 | Forward | GGGTCCCCAGTAAGATCCAG |
| BRAF  exon11 | Revers | CCCACTGTAATCTGCCCATC |
| KIAA1549  exon10 | Forward | TGGTGGTGATGGTGATTGTT |
| BRAF  exon9 | Revers | GGGGTAGCAGACAAACCTGT |
| CLIP2  exon8 | Forward | TGCTCAAGGCACAGCATGAGGAGT |
| NTRK2  exon12 | Revers | TCCCATTGGAGATGTGATGGAGTG |
| QKI | Forward | GCAGCTGATGAACGACAAGA |
| RAF1 | Revers | AGAACCACTCCAGCGTGACT |
| KANK1  exon2 | Forward | GACACTGCTGGCTGAGAACTAC |
| NTRK2  exon14 | Revers | AGTCATCATCATTGCTGATAAC |
| BRAF  exon11 | Forward | TAGTGAGCCAGGTAATGAGGCA |
| GANI1  exon1 | Revers | TGGAGCGGAGTAAGATGATCGA |
| BCAS1  exon11 | Forward | TGGACACGAACTCACTGCAGAATG |
| BRAF  exon9 | Revers | AGTGAGCCAGGTAATGAGGCAGGG |
