# Supplementary material for: Integrated genomic analysis reveals actionable targets in pediatric spinal cord low-grade gliomas
Source: Acta Neuropathol Commun. 2022 Sep 26;10:143. doi: 10.1186/s40478-022-01446-0 (PMC9513869; doi:10.1186/s40478-022-01446-0)
Supplement: Supplementary file 2 — Additional file 2: Table S2. Table showing the complete sLGGs cohort emphasizing the original histology before molecular pathology reevaluation, anatomical location, and molecular-biology data. [file 40478_2022_1446_MOESM2_ESM.pdf]

	Original histology	Localization in spine	Age at the diagnosis [years]	Survival status	Molecular alteration	*CDKN2A* deletion	Methylation class calibrated score (classifier v12.5)	Group by genetic alteration
sLGG_01	Pilocytic atrocytoma	C5 - Th1	15.45	Alive	*KIAA1549:BRAF ex16:ex9*	neg	Pilocytic astrocytoma, infratentorial 0.73	***KIAA1549:BRAF* common fusions (9pts)**
sLGG_02	Diffuse astrocytoma	C1 - C7	17.54	Alive	*KIAA1549:BRAF ex15:ex9*	neg	Difuse leptomeningeal glioneuronal tumour, MC1 0.99	
sLGG_03	Diffuse astrocytoma	MO - C5	1.82	Alive	*KIAA1549:BRAF ex15:ex9*	neg	Pilocytic astrocytoma, infratentorial 0.86	
sLGG_04	Diffuse astrocytoma	Th10 - L1	4.20	Alive	*KIAA1549:BRAF ex15:ex9*	neg	Pilocytic astrocytoma, infratentorial 0.97	
sLGG_05	LGG NOS	C3 - C6	2.57	Alive	*KIAA1549:BRAF ex15:ex9*	neg	Pilocytic astrocytoma, infratentorial 0.99	
sLGG_06	Pilocytic atrocytoma	Th2 - Th7	6.93	Alive	*KIAA1549:BRAF ex15:ex9*	neg	Pilocytic astrocytoma, infratentorial 0.53	
sLGG_07	Pilocytic atrocytoma	C5 - Th3	2.02	Alive	*KIAA1549:BRAF ex15:ex9*	neg	Pilocytic astrocytoma, infratentorial 0.94	
sLGG_08	Diffuse astrocytoma	holocord	15.63	Alive	*KIAA1549:BRAF ex15:ex9*	neg	Control tissue 0.86	
sLGG_09	Diffuse astrocytoma	C5 - Th9	12.12	Alive	*KIAA1549:BRAF ex15:ex9*	neg	Pilocytic astrocytoma, infratentorial 0.72	
sLGG_10	Diffuse astrocytoma	Th10 - L1	16.48	Alive	*KIAA1549:BRAF ex16:ex11*	neg	Difuse leptomeningeal glioneuronal tumour, MC1 0.25	***KIAA1549:BRAF* rare fusions (5pts)**
sLGG_11	Diffuse astrocytoma	conus	10.82	Alive	*KIAA1549:BRAF ex15:ex11*	neg	Pilocytic astrocytoma, infratentorial 0.98	
sLGG_12	Pilocytic atrocytoma	Th7 - Th10	4.19	Alive	*KIAA1549:BRAF ex13:ex11*	neg	Pilocytic astrocytoma, infratentorial 0.97	
sLGG_13	Pilocytic atrocytoma	C4 - Th4/5	1.58	Alive	*KIAA1549:BRAF ex13:ex11*	neg	not suficient material	
sLGG_14	Diffuse astrocytoma	C4 - Th2	4.67	Alive	*KIAA1549:BRAF ex13:ex9*	neg	not suficient material	

	Original histology	Localization in spine	Age at the diagnosis [years]	Survival status	Molecular alteration	CDKNA2 deletion	Methylation class calibrated score (classifier v12.5)	Group by genetic alteration
sLGG_15	Pilocytic atrocytoma	MO - C5	8.68	Alive	*KIAA1549:BRAF ex10:ex9*	neg	Pilocytic astrocytoma, infratentorial 0.35	***KIAA1549:BRAF* novel fusion (4pts)**
sLGG_16	Ganglioglioma	C1 - C4	2.24	Alive	*KIAA1549:BRAF ex10:ex9*	neg	Control tissue 0.48	
sLGG_17	Ganglioglioma	Th3 - Th7	10.58	Alive	*KIAA1549:BRAF ex10:ex9*	neg	Control tissue 0.96	
sLGG_18	Ganglioglioma	C2 - Th2	1.12	DOD	*KIAA1549:BRAF ex10:ex9*	neg	Desmoplastic infantile gagnliogl./astrocytoma 0.59	
sLGG_19	Pilocytic atrocytoma	Th1 - Th7	4.43	Alive	*BCAS1:BRAF*	neg	Pilocytic astrocytoma, infratentorial 0.74	**non-canonical *BRAF* fusions (2pts)**
sLGG_20	Pilocytic atrocytoma	Th4 - Th5	9.18	Alive	*GNAI1:BRAF*	neg	Pilocytic astrocytoma, infratentorial 0.42	
sLGG_21	Ependymom grade 3	Th12 - L3	3.13	Alive	*RAF1:QKI*	neg	Glioneuronal tumor, subtype A 0.99	**non-*BRAF* fusion (4pts)**
sLGG_22	Anaplastic astrocytoma	Th5 - L1	2.07	DOD	*CLIP2:NTRK2*	del	Anaplastic pilocytic astrocytoma, HGAP, 0.62	
sLGG_23	Ependymom grade 2/3	Th5 - Th12	2.04	Alive	*KANK1:NTRK2*	del	Pleomorphic xanthoastrocytoma, 0.90	
sLGG_24	Glioneuronal tumor	Th9 - Th12	3.41	Alive	KRAS Q61H	neg	Difuse leptomeningeal glioneuronal tumour, MC1 0.99	
sLGG_25	Diffuse astrocytoma	C4 - Th9	6.44	Alive	not suficient material	neg	not suficient material	N/A
sLGG_26	LGG NOS	holocord	12.33	DOD	not suficient material	neg	not suficient material	
