# Supplementary figures and images for: Integrated genomic analysis reveals actionable targets in pediatric spinal cord low-grade gliomas
Source: Acta Neuropathol Commun. 2022 Sep 26;10:143. doi: 10.1186/s40478-022-01446-0 (PMC9513869; doi:10.1186/s40478-022-01446-0)

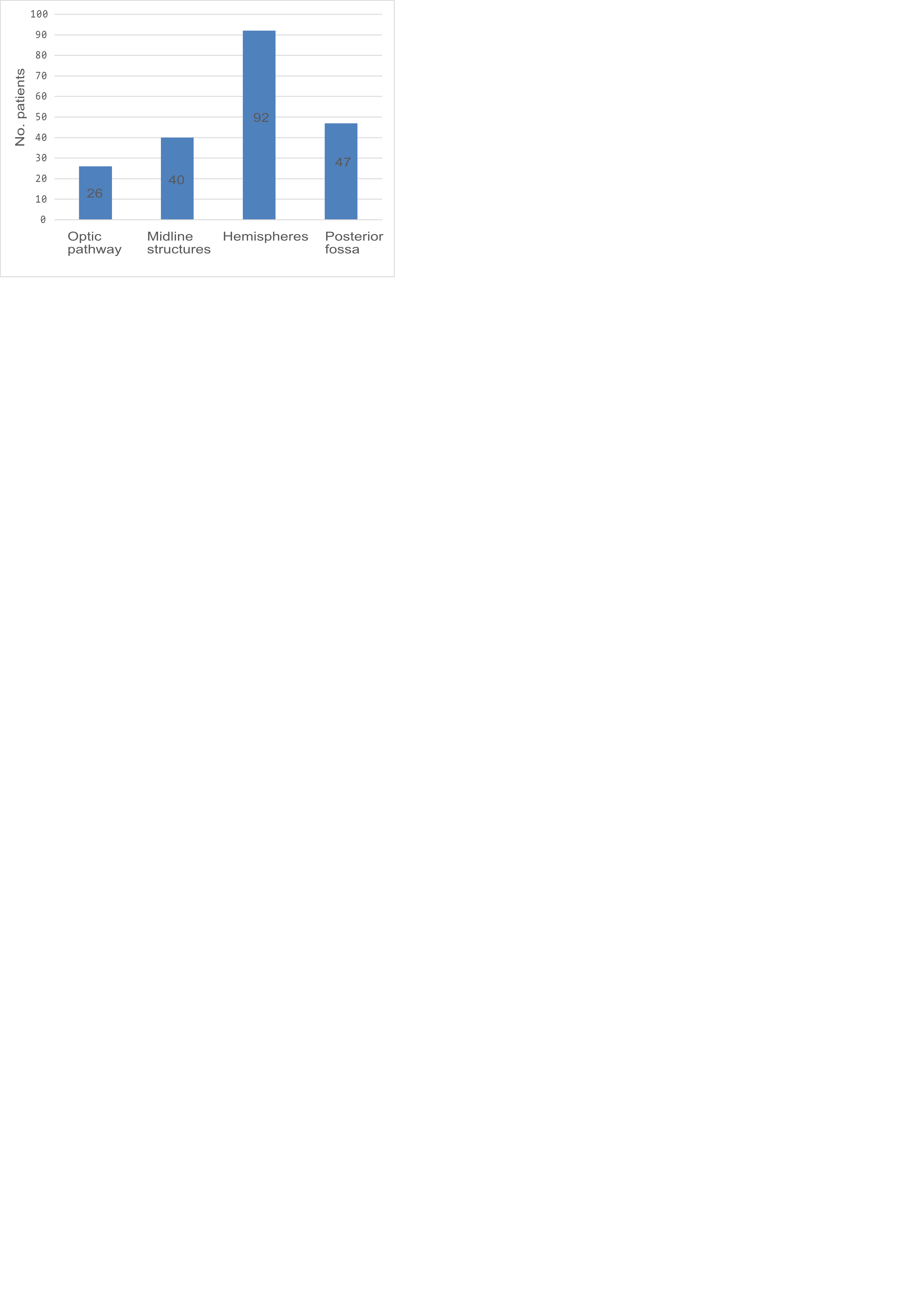

Supplement: Supplementary file 3 — Additional file 3: Fig. S1. A total number of pediatric intracranial LGG patients with known genetic alteration is dividedby anatomical location. Importantly, KIAA1549:BRAF ex9:ex10 variant fusion was detected solely in the upperspine. [file 40478_2022_1446_MOESM3_ESM.tiff]

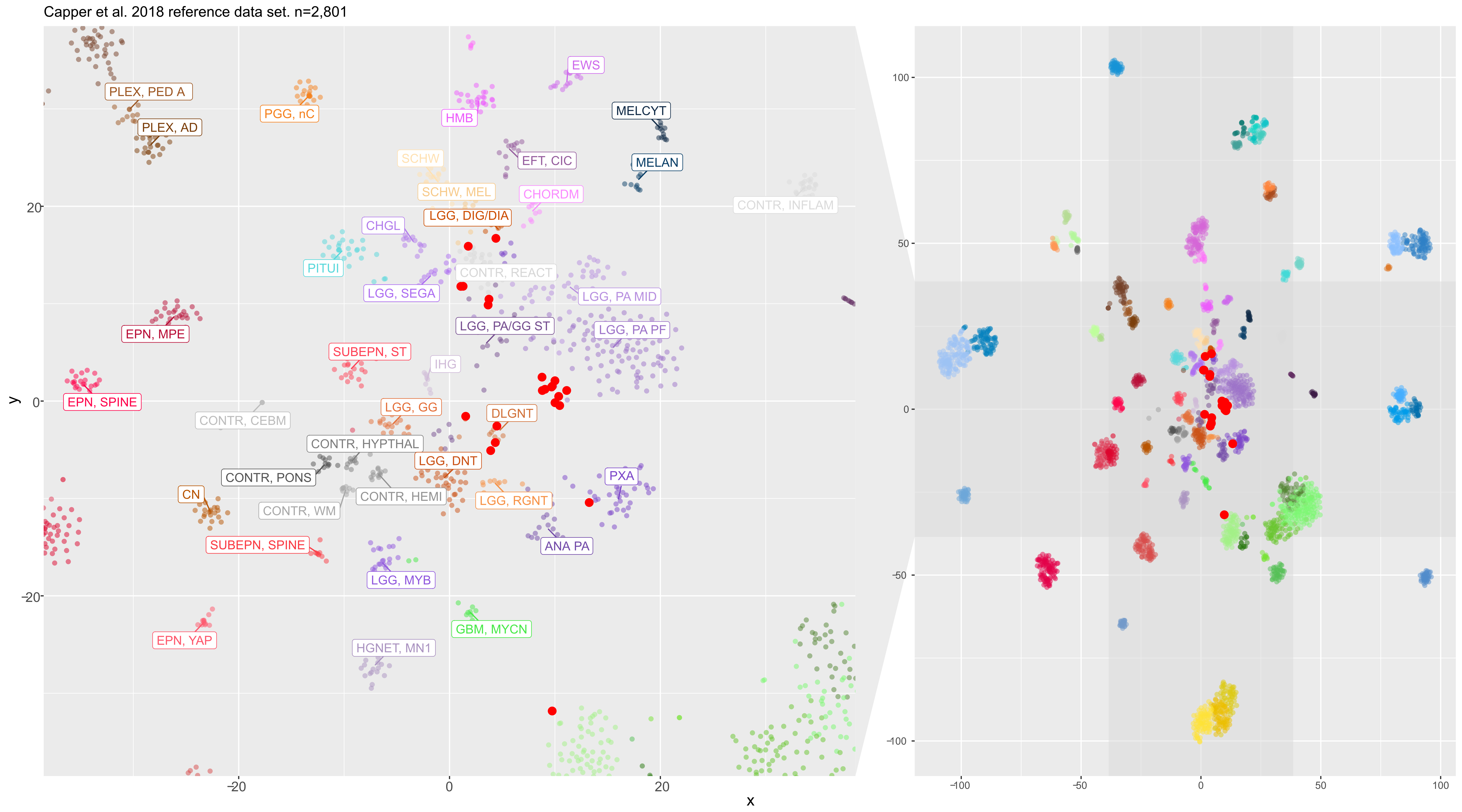

Supplement: Supplementary file 4 — Additional file 4: Fig. S2. T-SNE analysis displaying Prague samples (large red dots) among reference cohort samples. [file 40478_2022_1446_MOESM4_ESM.tiff]
